# Characterization of Muscle Spindle Afferents in the Adult Mouse Using an *In Vitro* Muscle-Nerve Preparation

**DOI:** 10.1371/journal.pone.0039140

**Published:** 2012-06-20

**Authors:** Katherine A. Wilkinson, Heidi E. Kloefkorn, Shawn Hochman

**Affiliations:** 1 Department of Physiology, Emory University School of Medicine, Atlanta, Georgia, United States of America; 2 Georgia Institute of Technology, Atlanta, Georgia, United States of America; University of Alberta, Canada

## Abstract

We utilized an *in vitro* adult mouse extensor digitorum longus (EDL) nerve-attached preparation to characterize the responses of muscle spindle afferents to ramp-and-hold stretch and sinusoidal vibratory stimuli. Responses were measured at both room (24°C) and muscle body temperature (34°C). Muscle spindle afferent static firing frequencies increased linearly in response to increasing stretch lengths to accurately encode the magnitude of muscle stretch (tested at 2.5%, 5% and 7.5% of resting length [Lo]). Peak firing frequency increased with ramp speeds (20% Lo/sec, 40% Lo/sec, and 60% Lo/sec). As a population, muscle spindle afferents could entrain 1:1 to sinusoidal vibrations throughout the frequency (10–100 Hz) and amplitude ranges tested (5–100 µm). Most units preferentially entrained to vibration frequencies close to their baseline steady-state firing frequencies. Cooling the muscle to 24°C decreased baseline firing frequency and units correspondingly entrained to slower frequency vibrations. The ramp component of stretch generated dynamic firing responses. These responses and related measures of dynamic sensitivity were not able to categorize units as primary (group Ia) or secondary (group II) even when tested with more extreme length changes (10% Lo). We conclude that the population of spindle afferents combines to encode stretch in a smoothly graded manner over the physiological range of lengths and speeds tested. Overall, spindle afferent response properties were comparable to those seen in other species, supporting subsequent use of the mouse genetic model system for studies on spindle function and dysfunction in an isolated muscle-nerve preparation.

## Introduction

Many conditions, including chronic muscle pain [1], [2], aging [3], [4], [5], [6], and diabetes [7], [8], involve changes in muscle spindle morphology or afferent activity. Skeletal muscle spindle afferent activity has largely been studied using *in vivo* preparations in the human [9], [10], [11], [12], [13], cat [14], [15], [16], [17], [18], [19], and rat [20], [21], [22]. Due to the small size of the mouse, direct recording of afferent cell bodies in the dorsal root ganglia [23], [24] and intra-axonal recording of afferents [25] have rarely been done *in vivo*. While *in vivo* preparations provide for studies of spindle function with intact circulatory and spinal circuitry, confounding variables include anesthetic choice, decerebration, and muscle perfusion status. An *in vitro* muscle-nerve preparation allows for the study of the direct response of a perturbation on the afferents and affords greater pharmacological and ionic control than *in vivo* preparations, though the ability to study integrated responses is sacrificed. Some characterization of muscle spindle responses in *vitro* has been done in the cat [26], [27], [28], [29], [30] and rat [31], [32], but to our knowledge, there have been no similar studies on mouse muscle spindle afferents.

This study describes the use of the isolated adult mouse extensor digitorum longus (EDL) muscle-nerve *in vitro* preparation as a model to study muscle spindle afferent activity. EDL is a fast twitch hindlimb calf muscle small enough to allow adequate diffusive oxygen supply at rest and with reasonable contraction duty cycles [33]. The isolated mouse EDL muscle is commonly used to study muscle contractile properties [34], [35], [36], [37] and the innervating nerve has been retained previously for electrically-evoked contractions [38], [39]. A similar *in vitro* preparation has been used in the rat to study mechanical responses of EDL group III and IV muscle afferents [40]. Here we characterize the response of muscle spindle afferents to a battery of ramp-and-hold stretches and sinusoidal vibrations at both 24°C (room temperature) and 34°C (body temperature) *in vitro*. As the mouse is the *de facto* mammalian model system for transgenic studies on disease mechanisms, the present description should provide an important baseline description of spindle function.

## Methods

### Ethics Statement

All procedures were approved by the Emory University Institutional Animal Care and Use Committee. Thirty-four C57BL/6 male, adult mice (average age 83.3±18.5 days, average weight 24.5±2.3 g) were used in this study.

### Muscle-Nerve Preparation

Animals were deeply anesthetized using isofluorane anesthesia and immediately decapitated and skinned. The legs were removed above the hips using large scissors and immediately placed in a dish of continuously oxygenated (95% O_2_, 5%CO_2_) low calcium, high magnesium artificial cerebrospinal fluid, containing in mM: 128 NaCl, 1.9 KCl, 1.2 KH_2_PO_4_, 26 NaHCO_3_, 0.85 CaCl_2_, 6.5 MgSO_4_, and 10 glucose (pH of 7.4). The deep peroneal branch of the sciatic nerve was dissected out and traced to the calf muscle EDL and all other branches cut. The tendons of the EDL were cut at the knee and ankle joints and the muscle and nerve removed and transferred to a 25 ml *in vitro* bath plate (809B-IV, Aurora Scientific, Inc.). The bath was perfused with oxygenated (100% O_2_) synthetic interstitial fluid containing in mM 123 NaCl, 3.5 KCl, 0.7 MgSO_4_, 1.7 NaH_2_PO_4_, 2.0 CaCl_2_, 9.5 NaC_6_H_11_O (sodium gluconate), 5.5 glucose, 7.5 sucrose, and 10 *N*-2-hydroxyethylpiperazine-*N′*-2-ethanesulfonic acid (HEPES) (pH 7.4±0.05) [41]. Experiments were done at either room temperature (24°C) or muscle body temperature (34°C). To heat the muscle and bath to 34°C, warm water was pumped into channels in the base plate of the dish and the reservoir of synthetic interstitial fluid was initially heated to 34°C and then wrapped in microwaveable clay heating pads to help maintain temperature throughout the experimental protocols. Bath temperature was continuously monitored throughout the experiment (BAT-12 Microprobe Thermometer and IT Series Thermocouple Probe, Physitemp) and the clay heating pads reheated as necessary.

**Figure 1:**
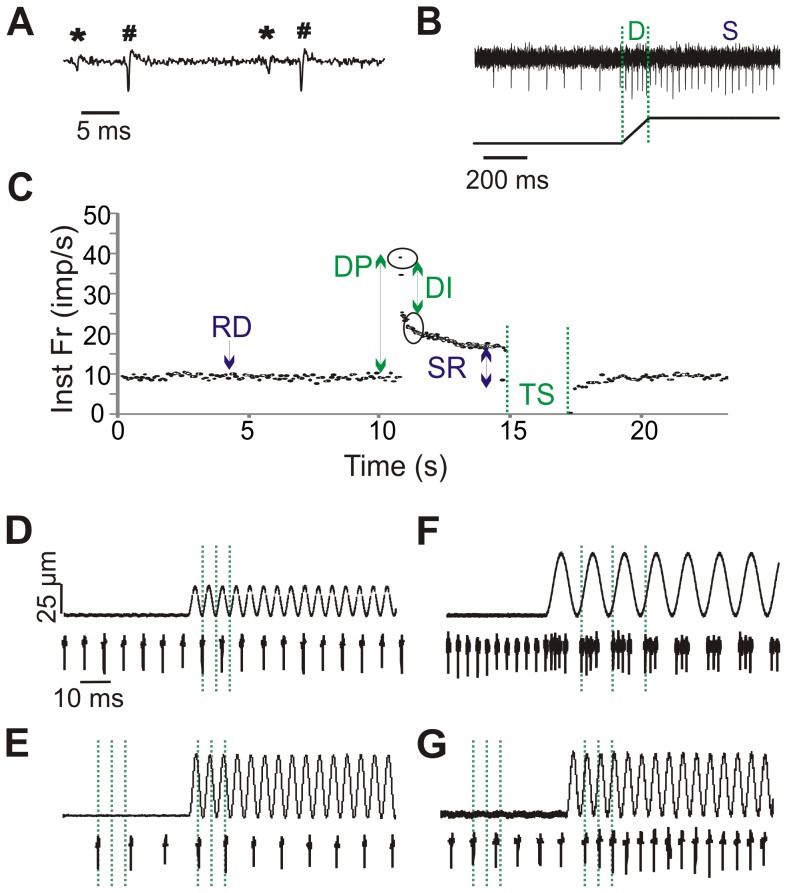
Data analysis methods. A. Raw waveform of 2 representative units (* and #). Spike2 software was used to identify unique waveforms based on shape. B. Response of 1 representative unit to ramp-and-hold stretch (length change of muscle shown below). The dynamic phase (D) of the stretch occurs during the ramp when the muscle is changing length. The static phase (S) of the stretch encompasses the time in which the length is held constant. C. Instantaneous frequency (Inst Fr) response to stretch of one unit. Arrows indicate time points where resting discharge (RD), dynamic peak (DP), dynamic index (DI), static response (SR), and time silenced (TS) are calculated. D-G: Afferent responses to vibration. D. No change in firing to a 25 Hz vibration. E. Entrainment at a subharmonic of 1:2 to a 25 Hz vibration. F. Multiple spikes per cycle at 10 Hz vibration. G. 1:1 entrainment at 25 Hz.

Both tendons of the EDL were tied with 5-0 nylon sutures and one end tied to a tissue post. The other end was tied to the lever arm of a dual force and length controller (300C-LR, Aurora Scientific, Inc). Suture length was minimized as much as possible, and compliance tests on the suture found that deformation was negligible over the forces studied. A bipolar glass suction electrode (diameter 25–50 µm) was placed on a portion of the cut end of the nerve and made tight-fitting via initial application of negative pressure. This electrode was used to record muscle spindle afferent activity. As shown in Figure 1A individual units were easily resolved due to large signal-to-noise ratios. Indeed, signal-to-noise ratios were large enough to discriminate group III/IV units in response to capsaicin (not shown), though because the amplitude of the spikes is dependent upon the unit’s distance from the electrode, amplitude was not a reliable method to classify afferents. Given this, it is unlikely that there was a sampling bias towards larger units that would more likely be Ia afferents. However, as the relative number of Ia vs. II afferents in EDL is unknown, a sampling bias would be anticipated to reflect their relative proportions. Previous work suggests that there is wide variation in the number of afferents innervating each spindle in a given muscle and across muscle and species types. In cats and rats, on average there is the same number of muscle spindle primaries as secondaries in most muscles [42]. If true for the mouse EDL, the present sample should contain a comparable number of Ia and II units. As there are ∼11 spindles in the mouse EDL muscle [43] recorded units (usually 1–2 per muscle and as many as 4 per muscle) would arise from a total population of ∼22 spindle afferents.

Resting length (Lo) of the muscle was determined by measuring the length at which maximum force following twitch contraction could be obtained following nerve stimulation. This was undertaken via electrical stimulation using the same suction electrode used for recording afferent activity (0.5 mA, 0.5 ms single stimulation pulse). As the optimal length determined by twitch contractions has been reported to be very close or identical to the optimal length determined by maximal isometric tetanic contractions [44], we used twitch contractions to prevent unnecessary muscle fatigue. All stretches and contractions were done at that resting length. Once resting length was determined, afferent activity was recorded from the nerve for at least 1 hour before the stretch and vibration protocols were started.

### Identification of Units as Muscle Spindle Afferents

In total 53 units were recorded and included in this study. The activity of a subset of the afferents (9 units at 34°C) was recorded during twitch contraction produced by platinum paddle electrodes placed on either side of the muscle. A submaximal twitch was produced with a 0.5 ms, 10 mA current once every 5 s for one minute. All included units tested in this manner exhibited a pause in firing during the twitch contraction characteristic of muscle spindle afferents [45], [46]. The stretch responses of these afferents were not significantly different from the population sample not tested with contraction, so it is unlikely that any non-spindle afferents like Golgi tendon organs were included in our sample.

### Stretches and Vibrations

Ramp-and-hold stretches and sinusoidal vibrations were delivered by a dual force and length controller (300C-LR, Aurora Scientific, Inc). Three lengths of stretch (2.5% Lo, 5% Lo, and 7.5% Lo) and three speeds of ramp (20% Lo/sec, 40% Lo/sec, and 60% Lo/sec) were used in combination for a total of nine different ramp-and-hold stretches in most animals (13 units at 34°C received stretches at 7.5% Lo, 8.5% Lo, and 10% Lo). Each stretch lasted for 4 sec and was performed at least 3 times with a rest period between each stretch of at least 45 sec. Four frequencies (10 Hz, 25 Hz, 50 Hz, and 100 Hz) and 4 amplitudes (5 µm, 25 µm, 50 µm, 100 µm) of vibrations were used for a total of 16 different vibrations. Each vibration was performed for 9 sec with at least 45 sec between each vibration.

### Verification of Muscle Health Using Contractions

Muscle health at 24°C was determined in a subset of animals following the stretch and vibration battery using maximal isometric tetanic contractions. Briefly, platinum paddle electrodes were placed on either side of the muscle and a 350 ms train of 1 ms pulses at supramaximal voltage were applied at frequencies between 25 and 200 Hz (Grass S88 Stimulator). Maximal isometric tetanic contraction was found with a pulse frequency of 150 Hz. A peak force of 23.4±6.0 N/cm^2^ (n = 6) was observed at 150 Hz, similar to previously reported values [36]. This suggests that the muscle was functioning physiologically throughout the course of the experiment.

### Data Analysis

Data were acquired using pClamp acquisition software (version 10.0; Molecular Devices) and analyzed offline using Spike2 software (CED). Individual units were discriminated using the Spike2 spike sorting algorithm and analyzed separately (Fig. 1A).

Instantaneous firing frequencies were calculated at four different stages of the ramp-and-hold stretch as depicted in Figure 1C and described in [21]. Resting discharge (RD) was calculated as the mean frequency during the 10 s before the start of the stretch and the dynamic peak discharge (DP) was the difference between the highest discharge frequency at the end of the ramp phase and the RD. The dynamic index (DI) was the frequency difference between the highest frequency of the ramp phase and the frequency 0.5 s after the end of the ramp [47], [48]. The static response (SR) was defined as the frequency difference between the firing frequency 0.5 s from end of the stretch and RD and static sensitivity is the SR divided by the magnitude of stretch [49]. The time that the unit was silenced (TS) following the release of the stretch was also determined.

The response of a unit to vibration was determined at all vibration frequencies and amplitudes. Units were classified as showing no change in firing to vibration (Fig. 1D), entraining to a subharmonic (i.e. 1:2, Fig. 1E), entraining in a x:1 manner (Fig. 1F), or entraining in a 1:1 manner (Fig. 1G) over the whole 9 s of vibration.

### Statistics

**Figure 2:**
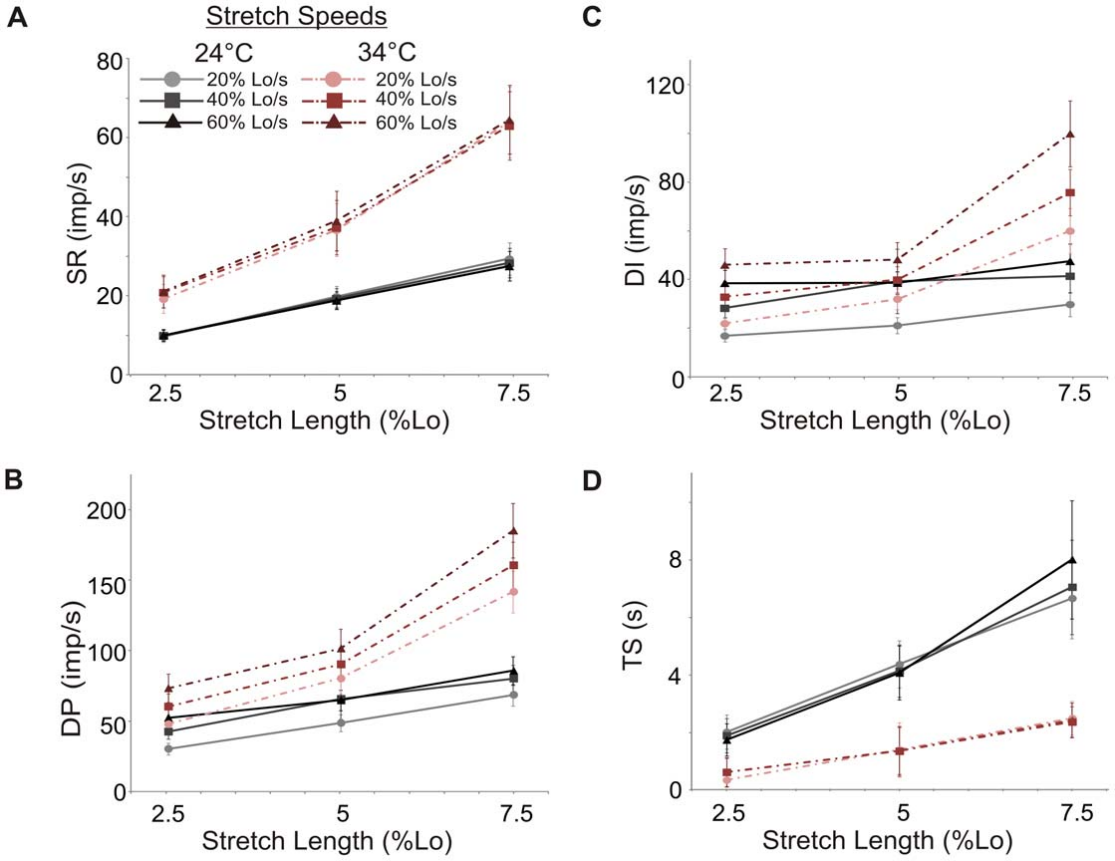
Effect of stretch length, speed, and temperature on spindle afferent firing. Response of 4 ramp-and-hold stretch variables to the three levels of stretch length (x axis) and 3 speeds of stretch as indicated in the legend atop A. Stretch speeds are denoted by separate lines at 24°C (solid black) and at 34°C (dashed colored). A. Static response (SR) B. Dynamic peak (DP) C. Dynamic index (DI) D. Time silenced (TS). Error bars indicate a 95% confidence interval.

Values are given as means ± SD. All differences were considered significant if p<0.05. Multivariate ANOVAs were used to compare ramp-and-hold stretch variables (i.e. DP, DI, etc) at the different stretch speeds, lengths, and temperatures. Tukey’s honestly significant difference (HSD) post-hoc test was performed to determine significant differences between the different levels of stretch speed and length. The error bars in Figure 2 illustrate 95% confidence intervals.

## Results

### Response to Ramp-and-Hold Stretch

In total, responses to ramp-and-hold stretch were recorded from 27 units from 21 animals at 24°C and 26 units from 21 animals at 34°C. Four of those units (from 3 animals) were recorded at both temperatures.

### Resting Discharge

Resting discharge was measured and used to compare changes in firing during stretch. As we set the length of our muscle to the length at which the largest force of twitch contraction could be elicited, we predicted that most spindle afferents would be within their operating ranges and so could respond to increases in length by increasing frequency and would likely have a resting discharge. Due to the fact that spindles are distributed throughout the muscle [43], some spindles were likely not as well aligned with the axis of tension and may not display baseline firing. As predicted, most units exhibited a resting discharge before stretch (23/27 units at 24°C and 23/26 units at 34°C). The resting discharge did not vary significantly over the 45 min it took to complete the battery of stretches at either temperature, suggesting that the spindle afferents were not undergoing time dependent changes in firing properties and that the muscle was returning to resting length following each stretch (RD at 15, 30, and 45 min at 24°C (imp/s): 11.8±5.7, 11.6±5.6, 12.1±8.2; 34°C: 32.0±17.6, 31.9±18.0, 32.4±17.2). Resting discharge was significantly higher at 34°C than at 24°C (32.3±17.3 imp/s vs. 11.9±6.3 imp/s, p<0.001), similar to the temperature dependence of firing frequency reported in cats [29].

### Relation between Stretch Length and Firing Frequency at Steady State

Muscle spindle afferents encode muscle length in their frequency of firing. At both temperatures, the static response increased with stretch length (24°C & 34°C p<0.001), but was not affected by stretch speed (24°C p = 0.22; 34°C p = 0.78; Fig. 2A). If the static response was normalized to the absolute stretch length (static sensitivity: SR/stretch length), values at all stretch lengths and speeds were the same at 24°C (i.e. the change in firing per mm of stretch was a constant value as expressed in imp/s/mm, 2.5% Lo: 29.0±19.2; 5% Lo: 28.2±16.4; 7.5% Lo: 27.5±16.6). At 34°C, there were no significant differences in static sensitivity between all stretch lengths except at the 5% Lo length as compared to 7.5% Lo (p = .01 in Tukey’s HSD post-hoc; 2.5% Lo: 59.3±36.7; 5% Lo: 54.6±33.0; 7.5% Lo: 65.1±40.0 imp/s/mm). Overall, once steady state firing is reached, mouse spindle afferents accurately and linearly encode the magnitude of muscle stretch over the lengths tested. Like resting discharge, the magnitude of the static response was higher at 34°C than at 24°C (p<.001; Fig. 2A).

### Measures of Dynamic Responses to Stretch

In addition to the static encoding of length changes, spindle afferents, especially primary afferents, can respond to dynamic length changes. At both temperatures, dynamic peak firing increased with increasing stretch length (p<0.001; Fig. 2B), and was higher at 34°C than at 24°C (p<0.001). While increasing the rate of speed also led to higher dynamic peaks at both temperatures (p<0.001), the 40% Lo/s and 60% Lo/s speeds were not significantly different from each other at 24°C (p = 0.26) nor were the 20% Lo/s and 40% Lo/s speeds at 34°C (p = 0.08).

Like dynamic peak, there was a main effect of both stretch speed and length on dynamic index (all p<0.001; Fig. 2C) and dynamic index was significantly higher at 34°C than 24°C (p<0.001). The dynamic index at all speeds and lengths was significantly different, except at 24°C, where the dynamic index trended towards a plateau at higher stretch speeds (all p<0.05 except 40% Lo/s vs. 60% Lo/s, p = 0.14, Tukey’s HSD post-hoc). This suggests that peak dynamic spindle responsiveness at 24°C occurs around 40% Lo/s (approximately 5.6 mm/s).

The time the unit was silenced following the release of stretch (TS) was always longer in 24°C as compared to 34°C (p<0.001; Fig. 2C). The time the unit was silenced following stretch increased with increasing stretch lengths at both temperatures (p<0.05 at all levels). However, unlike dynamic peak and dynamic index, time silenced was independent of stretch speed.

### Are Spindles Divisible into Primary and Secondary Units Based on Response Properties?

**Figure 3:**
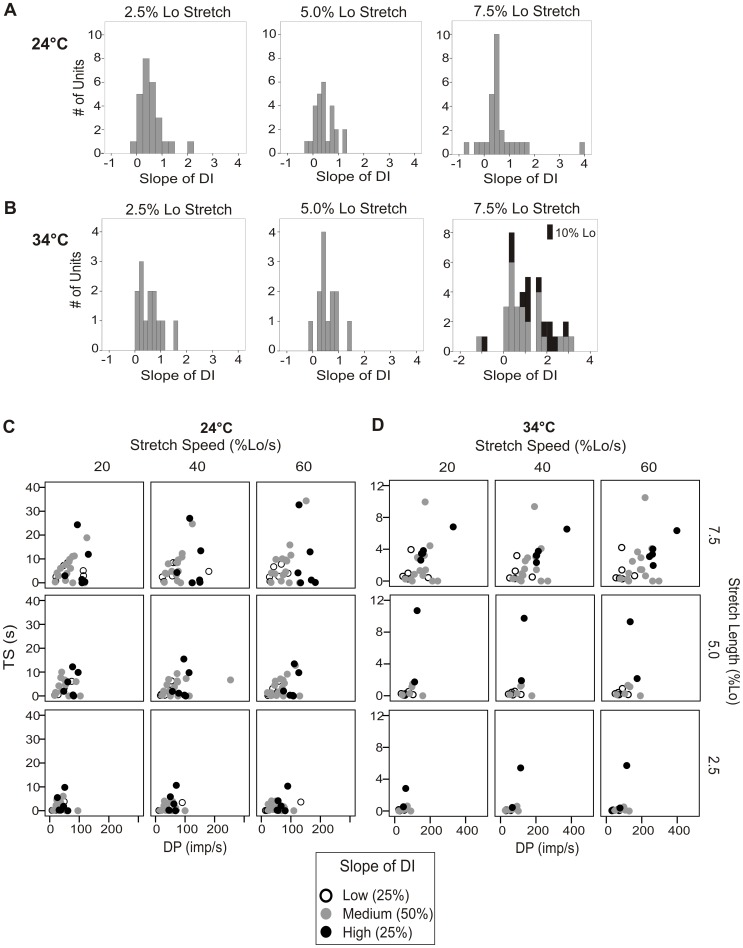
Spindle afferents could not be definitively classified using measures of dynamic sensitivity. A and B. The slope of the linear regression of DI over the 3 stretch speeds tested was calculated at all 3 stretch lengths and shown for 24°C (A) and 34°C (B). No bimodal distribution was observed, though there was the largest variation in response at the largest stretch length. The response of a subset of the animals (n = 13) to a larger stretch length at 34°C (10% Lo) is shown stacked in black associated with values obtained at 7.5% Lo. No additional separation was observed at even this extreme length. The DP vs. TS responses of the 25% of the units with the highest (open circles), middle 50% (gray circles), and bottom 25% (black circles) slopes of DI are plotted at 24°C (C) and 34°C (D).

The main reported difference between primary (Ia) and secondary (II) spindle afferents is the increased sensitivity to length changes of the primary afferents [50]. In de-efferented rats, the slope of the linear regression of the dynamic index over multiple stretch speeds was reported to yield a bimodal distribution that became more apparent with increasing stretch amplitude. The categorization of primary or secondary afferents based on the linear regression of a unit’s dynamic index coincided with the categorization based on its conduction velocity, which in the rat yields a distribution with two peaks, but is not completely free of overlap [21]. Here, we did not observe a bimodal distribution of the slope of the linear regression of dynamic index over the 3 stretch speeds tested at either temperature at any level of stretch (Fig. 3A–B). Similarly, the dynamic index, dynamic peak, or time the unit was silenced after stretch did not clearly separate the units into two groups (data not shown). To see if we could find clear differences at the extremes, we separated the units into the 25% with the highest slope of linear regression of dynamic index, middle 50%, and lowest 25%. When the dynamic peak and time silenced were compared between the top and bottom 25% groups, the high slope group had a higher dynamic peak, dynamic index, and time silenced than the low slope group. Moreover, if each unit’s dynamic peak was plotted against time silenced, a trend for the high slope of dynamic index units to have a higher dynamic index and time silenced was observed, especially at 34°C at the largest stretch length (Fig. 3C–D). Further testing of a subset of the units at 34°C (n = 13) at higher stretch lengths (8.5% Lo and 10% Lo) did not provide additional separation of units based on dynamic properties (see Fig. 3B stacked in black for 10% Lo stretch length).

### Response to Sinusoidal Vibration

Another measure of how sensitive spindle afferents are to dynamic length changes is how well they can entrain to vibrations. Responses to vibration were recorded from 13 units in 12 animals at 24°C and 15 units in 12 animals at 34°C. In total, the units were tested with 9 s of 16 different vibrations comprised of 4 different peak-to-peak amplitudes (5 µm, 25 µm, 50 µm, 100 µm) at 4 different frequencies (10 Hz, 25 Hz, 50 Hz, and 100 Hz).

**Figure 4:**
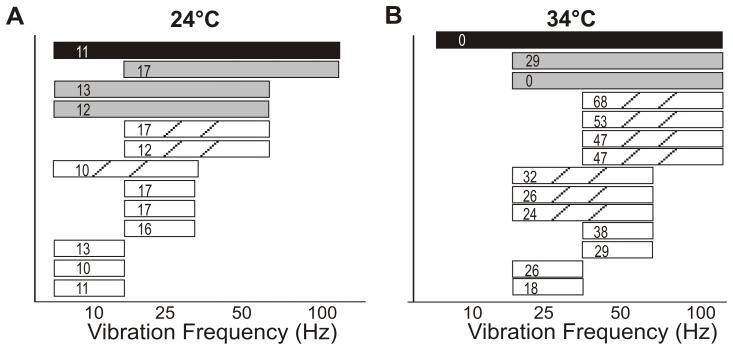
Frequency range of entrainment for individual units. Frequency entrainment ranges for individual units are shown at 24°C (A) and 34°C (B). Each bar represents an individual unit and covers the frequency range that unit could entrain to. The patterns of the bar signify the number of frequencies the unit can entrain to (black  = 4, gray  = 3, hatched  = 2, white  = 1). The unit’s baseline resting discharge is shown in Hz in the bar.

In response to vibration we observed a few types of characteristic responses (Fig. 1 D-G). Some units increased their firing in response to vibration, but did not follow a specific pattern. Some could not fire fast enough to entrain 1:1 but entrained to a subharmonic (i.e. 1:2, Fig. 1E). Entrainment to a subharmonic occurred at 25, 50, and 100 Hz at 24°C and was quite common, with ∼60% of the units in 100 Hz vibrations of amplitudes greater than 25 µm entraining to a subharmonic. At 34°C, subharmonic entrainment was rarely seen at 50 Hz and fairly common at 100 Hz (up to 60% of the units). Some units fired multiple times per cycle at the 10 and 25 Hz frequencies at 34°C (Fig. 1F). Entrainment to 10 Hz frequency in body temperature was rarely seen and would have required a large decrease in firing frequency from an average resting discharge of ∼32 imp/s. Perfect entrainment occurred when the unit fired 1:1 during the same time in the vibration cycle (Fig. 1G). Units could entrain to vibration by either increasing or decreasing their resting firing frequency as shown in Figure 4. Some units could entrain to frequencies both above and below their resting discharge.

The percentage of units that entrained to vibration is shown in Table 1. All units entrained 1:1 to at least 1 of the 16 vibrations tested. At both temperatures as the amplitude of vibration increased, the number of units that entrained to the vibration also increased (Table 1). Units had preferred frequencies of vibration and few units could respond to more than 2 different frequencies of vibration. At 24°C, 1 unit responded to all 4 frequencies, 3 units responded to 3 frequencies, 3 units to 2 frequencies, and 6 units only responded to one frequency (Fig. 4A). At 34°C, 1 unit could respond to 4 frequencies, 2 to 3 frequencies, 7 to 2 frequencies, and 4 to only 1 frequency (Fig. 4B). In general, units entrained to the frequencies nearest to their baseline frequency (shown in Fig. A–B), though the unit that had the highest range of response at both temperatures also had a relatively low baseline firing rate. Units at 24°C preferentially entrained to low frequencies of vibration (10 and 25 Hz) and at 34°C to the higher frequencies (especially 50 Hz). At 34°C, only one unit entrained 1:1 to a 10 Hz vibration, but the percentage of units that entrained with multiple spikes per cycle is shown in parentheses in Table 1.

**Table 1:** Percentage of units that could follow vibration 1:1 at 24°C and 34°C.

	24°C				34°C			
	10 Hz	25 Hz	50 Hz	100 Hz	10 Hz	25 Hz	50 Hz	100 Hz
**5 µm**	25%	0%	0%	0%	0% (0%)	13% (0%)	7%	0%
**25 µm**	50%	25%	0%	0%	7% (13%)	40% (0%)	47%	0%
**50 µm**	58%	42%	8%	0%	7% (27%)	53% (13%)	80%	20%
**100 µm**	54%	77%	38%	15%	0% (73%)	47% (26%)	87%	50%

Rows represent different amplitudes of vibration (µm) and columns different frequencies of vibration (Hz). Most vibrations tallied the response of 13 units at 24°C and 15 at 34°C, though a few had one less unit due to technical issues. At 34°C, some units fired multiple times per cycle at 10 and 25 Hz (% of units shown in parentheses). Two units were sampled at both 24°C and 34°C.

**Figure 5:**
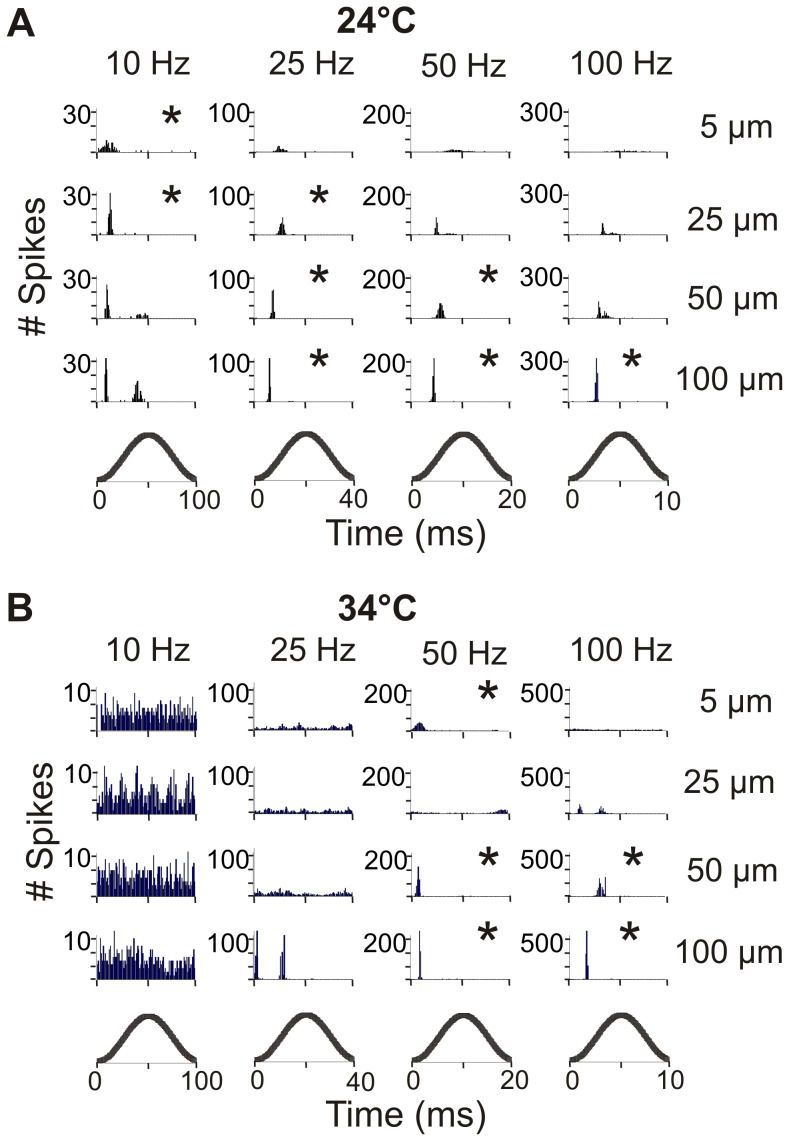
Response to vibration of one unit at 24°C and 34°C. Number of spikes at a given time over the vibration cycle for all 16 vibrations at 24°C (A) and 34°C (B). Bottom traces show the stage of vibration cycle. Amplitude of vibration increases down the column and frequency increases left to right. * denotes 1:1 entrainment of the unit.

When a unit entrained to vibration, it usually showed a very narrow time range to spike during the stretching phase of the cycle. An example is shown in a unit measured in both room and body temperature (Fig. 5). A histogram showing the number of spikes at a given point in the vibration is shown for each vibration. The time axis is cycle normalized, so the higher frequency vibrations are completed over a shorter time than the lower frequency vibrations. If a unit entrained to vibration, it fired preferentially near the beginning of the vibration in the stretch phase of the cycle.

Compared to the ramp-and-hold tests, sinusoidal vibrations tested dynamic responsiveness at much smaller length changes. Units that entrain to the lowest amplitude of stretch or the highest frequency of vibration presumably have the greatest sensitivity to length changes, and may correspond to those units that have high measures of dynamic sensitivity in the ramp-and-hold stretches (DP, DI, TS, or high linear regression slopes of DI). We observed no such relation. Similarly, units that entrain to several vibration frequencies may be considered to have the greatest dynamic response range, yet again there was no clear correlation to their dynamic measurements during ramp-and-hold stretch. Overall, the responses to vibration did not overtly differentiate units into two categories that may identify spindle primaries from secondaries.

## Discussion

### Validation of Model

We have developed a novel preparation that will allow for the further study of stretch sensitive afferents in the adult mouse. Our preparation modifies the isolated muscle preparation already in use by many investigators studying contractile properties of muscle [34], [35], [36], [37] to include the recording of sensory information from an attached nerve. Muscle health was normal over the course of our recordings as measured by the maximum contractile force the muscle could generate. Additionally, the resting discharge did not differ significantly throughout the course of the experiment at both temperatures, suggesting that there were no time dependent changes in the unit’s firing properties over the time course of the experiment.

To be consistent between animals and compare our results to that of other species, we determined the length at which the muscle could produce the maximum force of contraction and set that as resting length, or Lo. In some animals we also measured the muscle length when the knee and ankle joints were both set to 90° angles [51] and found this measure to be very similar to Lo. As expected, most spindle afferents at Lo had a resting discharge and could respond to both increases and decreases in length by changing their firing rate.

While we cannot directly rule out the possibility that recorded units include Golgi tendon organs (Ib) or stretch sensitive group III or IV afferents in all cases, we are confident that observed responses arose from muscle spindle afferents. Ib afferents do not typically show a response to stretch unless it is a very large stretch (e.g. greater than the physiological range) [52], [53]. Moreover, we evoked contractions in a subset of units (9/26 at 34°C), and observed the pause in firing characteristic of spindle afferents rather than the increase in firing that is found with Ib Golgi tendon organ afferents [45], [46], [54], [55]. Stretch sensitive group III or IV units are reported to have very low firing rates (∼0.2 imp/s) and only slightly increase their firing in response to stretch to ∼1.2 imp/s [56]. While we found some units with no baseline firing frequency, they increased their firing rates to >30 imp/s during stretch. Lastly, the observed unit responses comprised a continuous distribution with small standard error which supports sampling from a uniform afferent fiber population.

### Ramp-and-Hold and Vibration Responses are Comparable to Other Species

Many of the basic responses to stretch observed in our mice were similar to those reported in other species. The resting discharge we measured in the mouse is similar to that reported in rat and cat. In de-efferented *in vivo* rats at 34°C and a muscle length of 20% greater than the minimum length of the soleus muscle, resting discharge was reported to be ∼25 imp/s [21]. This is lower than that of our mouse EDL afferents (∼32 imp/s) that may have been held at a slightly greater tension. In isolated cat tenuissimus spindles at 34°C, firing frequencies compare well to ours, with resting discharges of ∼25 imp/s for primary units and ∼38 imp/s for secondary units. Similarly, their reported resting discharges at 24°C of ∼13 imp/s in primary units and ∼20 imp/sec in secondary units [29] are similar to our room temperature values of ∼13 imp/s. The static response to stretch in our units was constant and not dependent on speed of stretch (Fig. 3A), similar to what was observed by Matthews (1963). This suggests that once a relatively steady state has been reached, mouse spindle afferents are accurately and linearly encoding the magnitude of muscle stretch. The static response per mm of stretch in our mice (∼28 imp/s/mm at 24°C and ∼60 imp/s/mm at 34°C) was greater than values reported for de-efferented *in vivo* rats (∼17 imp/s/mm) [57] and cats (∼8 imp/s/mm in primary afferents and ∼4 imp/s/mm in secondary afferents) [14]. This increased static sensitivity in the mice is likely due to the smaller size of the muscle and smaller total range of motion. The dynamic response to stretch observed in mice was also similar to that reported in other species. Peak firing (DP) occurred during the ramp phase of stretch and the firing rate adapted to a relatively steady state 3.5 s after the final length was reached. The dynamic peak and dynamic index were increased by both stretch length and speed (Fig. 3 B-C). This is similar to what has been observed in rats and cats [21], [29].

The range of vibration frequencies and amplitudes tested demonstrated that as a population mouse spindle afferents report a large dynamic range of muscle stretch. This range of firing frequency entrainment is also seen in cats, rats, and humans [21], [26], [58], [59], supporting common mechanosensory encoding mechanisms across species. Though we did not determine the minimal amplitude or maximal frequency entrainment response of the population, overall, very few units could entrain to the vibrations of the lowest amplitude (5 µm) or the highest frequency (100 Hz). Most units entrained to vibrations of larger amplitudes and at frequencies near their resting discharge rate.

### Temperature Effects

In cat and frog spindles, resting spindle discharge increased with increasing temperature [29], [60]. Conversely, cooling can decrease firing frequency in humans and cats [61], [62]. The temperature differences in resting discharge we observed in our mice are thus consistent with those observed in other species. These temperature differences in response properties are consistent with the fact that higher temperatures increase the rate of ion channel gating [63], [64] and can also increase channel conductance [65]. Similarly, as seen in the isolated cat spindle [29], the dynamic peak, dynamic index, and static response values were higher at 34°C. Moreover, at the higher stretch speeds, only the units at 34°C continued to show an increase in the dynamic index (Fig. 2C). At 34°C, units had a shorter silent period (TS) after stretch but units at both temperatures showed a linear increase in TS to stretch length with no effect of stretch speed. In summary, spindles showed similar responses to stretch at both temperatures, with greater firing rates and magnitudes of change occurring at 34°C.

Regarding vibratory stimuli, units were more likely to entrain to vibrations of lower frequencies at 24°C (10 or 25 Hz) and higher frequencies at 34°C (50 or 100 Hz). This is not surprising as units tended to entrain near their resting discharge which was much lower at 24°C (∼13 imp/s) than 34°C (∼32 imp/s). There were, however, units at 24°C that could entrain to the highest frequency and units at 34°C that could follow a 10 Hz vibration with one or multiple spikes per cycle.

### Units Could Not be Definitively Classified as Primary or Secondary

Sensory endings innervating the muscle spindles are typically classified into two types of units, primary (Ia) and secondary (II), based on their anatomical location and functional responses to stretch. Primary axons are larger and have a faster conduction velocity [66], [67], that in cats can be used to differentiate primary and secondary endings [however see [58], [68]]. In mouse, at 24°C with a nerve distance twice as long as ours (∼10 mm), conduction velocity could not distinguish between group I and II afferents [69], and thus could not be used in our preparation to categorize units.

Functional measures of a spindle afferent’s response to stretch and vibration in humans and cats can generally be used to separate primaries and secondaries [9], [29], [47], [70], though in rats it is less clear, with one group reporting that 34% of their stretch sensitive units were not classifiable [71]. In de-efferented rats *in vivo*, the slope of the linear regression of the dynamic index during different stretch speeds produced a bimodal distribution of stretch sensitive units that closely matched the classification based on conduction velocity [21]. We did not see a bimodal distribution of dynamic index slope in our mice at any length tested (Fig 3 A-B). The units examined here in mouse exhibited a fairly continuous range of responses for all parameters examined and no combination of factors cleanly separated responses into two discrete groups. However, the range in population responses for almost all variables increased with increasing stretch lengths (Fig. 3D). Recordings from identified spindle primaries and secondaries may be required to generate classification criteria for unambiguous unit determination.

Alternatively, spindle afferent innervation patterns during rodent development may help explain the difficulty in unit classification. In the cat, primary fibers innervate the developing spindle before the chain fibers have formed, whereas the secondary endings innervate the spindle afterwards. In the rat, the time course for innervation is much shorter and many afferents have both primary and secondary-like terminals and more spindles in the rat contain multiple primary units than the cat [42], each of which may respond to stretch differently depending on the types of intrafusal fibers they contact. This allows for an anatomical explanation for the large number of units in the rat that cannot be definitively characterized as primary or secondary. While mouse spindle anatomy has not been investigated thoroughly, the observed non-bimodal distributions of stretch responses would be consistent with innervation patterns similar to that observed in the rat. Thus, there may be no consistent way to separate units into primary and secondary, or a third hybrid afferent population may emerge with response properties characteristic of both units – primary/secondary afferents. Future studies are necessary to determine if this prediction is correct.

### Significance and Future Studies

We described the population response to ramp-and-hold and sinusoidal vibratory stretch of spindle afferents from the EDL muscle of adult mice, including temperature dependence. These responses have been shown to be similar to rats, cats, and humans [29], [61], [62]. Due to the many available transgenic mouse lines and the ease of drug application in an *in vitro* preparation, we forward this system as well-suited to investigate spindle firing properties including their dysfunction and prospective therapeutic interventions in disease states.
